# Cystic appearance of low-grade endometrial stromal sarcoma in the right atrium: case report

**DOI:** 10.1186/1476-7120-9-23

**Published:** 2011-08-24

**Authors:** Cristina L Wood, James Sederberg, Paul Russ, Tamas Seres

**Affiliations:** 1 Department of Anesthesiology, University of Colorado Hospital, 12631 E. 17th Avenue, Aurora, 80045, USA; 2 Department of Anesthesiology, VA Medical Center, 1055 Clermont St.Denver, 80220, USA; 3 Department of Radiology, University of Colorado Hospital, 12631 E. 17th Avenue, Aurora, 80045, USA

**Keywords:** right atrial mass, intracardial cyst, low-grade endometrial stromal sarcoma, intraoperative transesophageal echocardiography

## Abstract

A 71-year-old woman presented with a right adnexal solid mass invading the right gonadal vein and inferior vena cava up to the hepatic veins revealed by CT and confirmed by MRI. A thin-walled cyst and a solid mass were unexpectedly found in the right atrium by transesophageal echocardiography (TEE) in the operating room. Using color Doppler and air bubbles as contrast material a circumscribed cyst was confirmed and localized close to the IVC. The cyst was connected to the mass in the inferior vena cava. The tumor, including the cyst, was removed without using cardiopulmonary bypass and described as a low-grade endometrial stromal sarcoma, a rare slowly growing tumor. This is the first TEE description of endometrial stromal sarcoma manifesting as a right atrial cyst.

## Background

The occurrence of a right atrial cyst is very rare. The differential diagnosis includes hydatid, blood and bronchogenic cysts. Primary tumors with cystic appearance can be rhabdomyoma or angiosarcoma [1,2]. Most of the secondary tumors which reach the heart through the inferior vena cava and invade the right atrium, such as renal and hepatocellular carcinoma, uterine leiomyoma or Wilms tumors, appear as solid masses [1]. In a case report, uterine leiomyoma presented as a right atrial cyst and caused recurrent episodes of sudden lightheadedness and profound weakness [3].

Low-grade endometrial stromal sarcoma (ESS) is a rare tumor, comprising only 0.2% of all uterine malignancies. Although the overall 5-year survival rate for low-grade ESS is higher than 80%, about 50% of the patients show tumor recurrence mostly after a long latency period. Invasion of the great vessels and the heart by this tumor is rare [4]. This is the first report of a low-grade ESS invading the inferior vena cava and ending in a cyst and a solid mass in the right atrium. The presence of the right atrial cyst was an unexpected finding by intraoperative transesophageal echocardiography (TEE), following diagnostic CT and MRI studies, supporting the role of TEE in diagnosing intra-atrial cysts.

## Case Report

**Figure 1:**
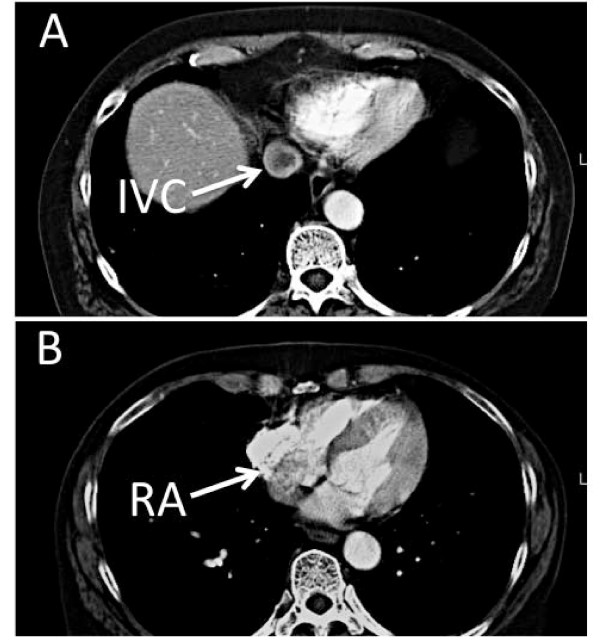
**Visualization of the IVC tumor using contrast enhanced axial CT. **Panel A: Dynamic contrast-enhanced axial CT scan through the suprahepatic IVC demonstrates central filling defect consistent with tumor or thrombus. Panel B: At an adjacent axial level, CT scan demonstrates a negative filling defect within the right atrium, suggesting the presence of an intra-atrial mass but the image is not conclusive for a cyst in the right atrium especially in comparison with the TEE images. IVC: inferior vena cava, RA: right atrium.

A 71-year-old female presented with several weeks of fatigue. Her pertinent medical history was significant only for a hysterectomy and oophorectomy in the 1980's for menorrhagia. A surveillance CT scan of a right lower lobe lung mass revealed a 3.6 × 2.5 cm right adnexal solid mass involving her right gonadal vein with extension into her inferior vena cava (IVC). A repeat chest CT, performed at an outside facility, reported that the mass extended up to the IVC-RA junction, with no intracardiac presence (Figure 1, Panel A and B). Based on the CT finding an abdominal MRI was performed where the mass appeared to propagate up to the confluence of the infrahepatic veins with unclear level of termination and based on the CT result no effort was made to study the right atrium. The patient did not have any renal, bowel or cardiac compromise at the time of presentation.

**Figure 2:**
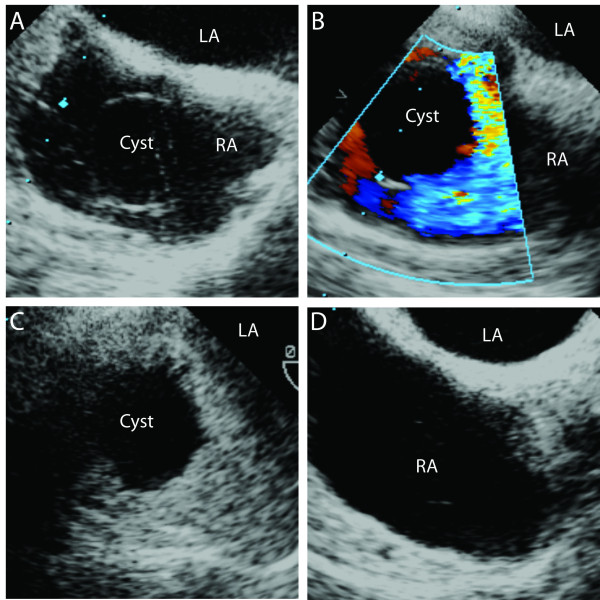
**In a modified midesophageal bicaval view, a cyst was seen in the right atrium**. Panel A: A cystic structure in the RA with a thin wall resulting in difficult visualization. Panel B: Color Doppler was used to confirm the existence of the cyst close to the IVC. Panel C: Bubble study, used to exclude PFO, confirmed the borders of the cyst and its position close to the IVC intersection. Panel D: A completely empty RA after the removal of the tumor. RA: right atrium, LA: left atrium, IVC: inferior vena cava.

**Figure 3:**
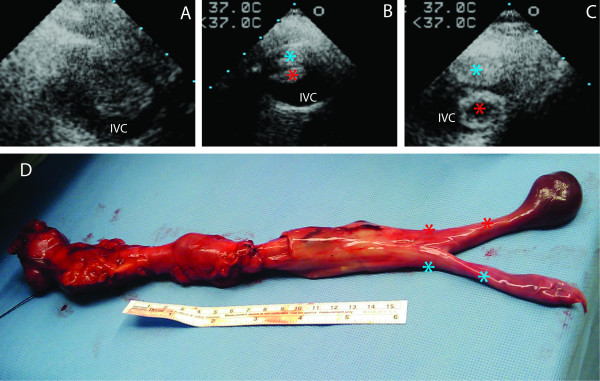
**A low-grade endometrial stromal sarcoma tumor divided into a solid and a tubular part**. The solid part ended in a solid head and the tubular part ended in a cyst. Utilizing TEE, each individual part of the tumor can be seen on Panel A, B and C located in the IVC.  Panel A: A solid tumor can be seen in the IVC.  Panel B: The tumor started dividing into two parts. The origin of the solid (blue asterisk) and tubular (red asterisk) part can be seen.  Panel C: Close to the IVC-RA intersection the tumor divided into a solid (blue asterisk) and a tubular (red asterisk) part.     Panel D: The removed tumor divided into a solid (blue asterisk) and tubular (red asterisk) neck and a solid and cystic head.  IVC: inferior vena cava, IVC-RA: inferior vena cava-right atrium.

Resection of the tumor was planned via laparotomy and sternotomy with possible cardiopulmonary bypass (CPB). Intraoperative TEE was performed after induction of general anesthesia. Using a slightly modified transesophageal bicaval view a cyst was seen unexpectedly in the right atrium (RA) with a very thin wall (Figure 2, Panel A). The existence of the cyst was confirmed by color Doppler and a bubble study where the bubbles surrounded the cyst (Figure 2, Panel B and C). Both studies suggested that the cyst was separated from the atrial walls and positioned close to the intersection of the IVC. The TEE probe was advanced in the direction of the IVC, where a mass was appreciated filling up about 50% of the lumen (Figure 3, Panel A). Moving the probe closer to the intersection of the IVC and RA, the mass was divided into a solid and a tubular part (Figure 3, Panel B and C). In the RA the cyst was detected again seemingly in continuation with the tubular part of the mass and the head of the solid part was sitting at the inter-atrial septum (Figure 3, Panel C and Additional File 1). The rest of the TEE examination was normal.

The tumor was removed intact via the IVC using TEE guidance without requiring CPB. After removal of the mass, the TEE showed no residual tumor and normal cardiac function (Figure 2, Panel D). The removed tumor divided to a cystic and solid head (Figure 3, Panel D). The TEE findings matched well with the tumor in the IVC and in the RA (Figure 3, Additional File 1). The patient tolerated the surgery well. The pathological review described the mass as a low-grade endometrial stromal sarcoma (ESS) with a cyst located at the cephalad portion of the tumor.

## Discussion

Transesophageal echocardiography can be critical in discovering, evaluating and localizing a right atrial mass. The differential diagnosis of right atrial masses ranges from congenital malformations, primary or secondary, benign or malignant tumors, thrombi and cysts. Congenital malformations include Eustachian valve, prominent crista terminalis or lipomatous hypertrophy [5,6]. A primary right atrial tumor can be a myxoma, rhabdomyoma, or angiosarcoma [1,7,8]. Secondary tumors can reach the heart through the inferior vena cava and invade the right atrium as solid masses, such as renal and hepatocellular carcinoma, uterine leiomyoma or Wilms tumors [1]. Among right atrial masses, right atrial cysts are rare and may represent hydatid, blood, bronchogenic cysts, cardiac varices or right coronary artery aneurysms [1,9,10]. Based on the appearance and location of the right atrial cysts, echocardiography helps in the differential diagnosis as follows:

A hydatid cyst in the right atrium is very rare and can appear as a multi-cystic mass with septations, calcifications and daughter cysts attached to the inter-atrial septum [11]. Multiple hydatid cysts can be close to the inferior and superior vena cava [12]. Blood cysts are congenital echolucent blood-filled nodules located on the endocardium, particularly along the lines of closure of heart valves [13]. Mobile oval blood cyst can be attached to the right atrial wall causing right coronary occlusion or as a round cyst attached to the inter-atrial septum with a thin, demarcated wall and echolucent core [14,15].

Intra-cardiac bronchogenic cysts are very rare. They appear as single thin walled homogenous cysts, similar to blood cysts [16]. They can be seen as intra-septal cystic masses on the right aspect of the inter-atrial septum causing complete AV block or as right atrial cysts attached to the inter-atrial septum [17,18].

Cardiac varix can be connected to the right atrial free wall as a large cystic lesion [10]. A right coronary aneurysm can appear as a cyst in the right atrium attached to the anterior wall with demonstrated flow within the mass by color Doppler [16]. A septal aneurysm [19], atrial thrombi [20], rhabdomyoma [1], myxoma [21] or angiosarcoma [2,8] might show cystic appearance also. Right atrial cysts associated to a secondary tumor can be diagnosed by the identification of the location and nature of the primary tumor.

In this case report, ESS as a secondary tumor invaded the IVC and RA. In our patient a thin-walled RA cyst was an unexpected finding as a continuation of an invading tumor not initially appreciated on CT. A retrospective review of the images did not indicate the presence of a RA mass at first. Even after knowing the presence of a cyst, a more detailed examination of the images of the RA raised the suspicion for an intra-atrial mass, but was not conclusive for an intra-atrial cyst (Figure 1, Panel B). The negative readings on CT are partially due to the differential composition of the solid tumor head versus the fluid filled cyst, as well as the mixing artifact secondary to the contrast load in the right atrium. This raises awareness to the need for additional analysis of radiological images in patients with IVC tumors, as well as the importance of the echo studies in confirming the intracardiac presence of these tumors.

Using color Doppler and air bubbles as contrast material a circumscribed cyst was detected and localized close to the IVC. Further TEE examination revealed that the tumor was divided into a solid and tubular part in the IVC and the cyst was connected to the tubular part. Two case reports with leiomyoma described similar structure where the cyst was connected with a tubular structure [3,22]. A literature review showed that low-grade ESS invaded the IVC in 19 patients. It extended into the right heart cavities in 9/19 patients (4 into the RA, 3 into the right ventricle and 2 into the pulmonary artery) [1]. No cystic appearance of ESS was described in these cases. There is only one case report in which an intraabdominal ESS had cystic manifestation [23]. This is the first case report of cystic appearance of low-grade ESS in the RA.

The clinical presentation of secondary tumors invading the IVC and RA includes signs and symptoms secondary to obstruction to forward flow in the right side of the heart. The most common clinical picture may present as right heart failure syndrome, syncopal episodes or both [3]. The presentation of ESS was unique in our case considering that the patient was essentially asymptomatic with an extended invasion into the IVC and RA. In addition, the recurrence of the tumor was detected more than 20 years after hysterectomy. This latency period was also seen in a case report of a low-grade ESS invading the IVC, the RA, the RV and obstructing the right ventricular outflow track 33 years after hysterectomy [24].

Given the high recurrence rates of ESS and the potential for aggressive invasion and progressive obstruction of the IVC, right heart surgical resection is the best treatment option [4]. Surgical resection of most of these tumors with cardiac invasion required CPB. In the present case, the tumor was resected through the IVC without CPB, using TEE to guide and evaluate the excision process.

We believe that ESS with intracardiac invasion should be considered in the differential diagnosis of intraatrial cystic masses.

## Consent

Written informed consent was obtained from the patient for publication of this case report and any accompanying images. A copy of the written consent is available for review by the Editor-in-Chief of this journal.

## Competing interests

The authors declare that they have no competing interests.

## Authors' contributions

CW collected the references and wrote the manuscript. JS contributed in writing the manuscript, making figures and revision of the paper. PR reviewed the CT and MRI images and helped to incorporate the data into the manuscript. TS initiated the paper and mentored the writing of the manuscript. All authors report no conflicts of interest and approved the final manuscript.

## Supplementary Material

Additional file 1 **TEE movie clip showing the ESS tumor present in the right atrium**. Movie clip showing the solid head of the tumor was sitting at the inter-atrial septum. The cystic head floated freely in the RA and connected to the tubular part of the mass in the IVC. RA: right atrium, IVC: inferior vena cava. Click here for file
